# Focus on the Brain: HIV Infection and Alcoholism: Comorbidity Effects on Brain Structure and Function

**Published:** 2010

**Authors:** Margaret J. Rosenbloom, Edith V. Sullivan, Adolf Pfefferbaum

**Keywords:** Alcohol abuse, alcoholism, risk factors, human immunodeficiency virus, acquired immune deficiency syndrome, brain, brain function, brain structure, neuropathology, magnetic resonance imaging, diffusion tensor imaging

## Abstract

Both HIV infection and alcohol abuse have negative effects on the brain, with some unique to each condition and others shared by both conditions. Investigators have used magnetic resonance imaging to study the size and integrity of various brain structures in participants with alcoholism, HIV infection, or both conditions and in healthy control subjects. In these studies, alcoholics exhibited enlarged, cerebrospinal fluid-filled spaces (i.e., ventricles) as well as tissue shrinkage in various brain regions (e.g., the corpus callosum and frontal cortex), whereas study participants with asymptomatic HIV infection showed few abnormalities. Those with both HIV infection and alcoholism also had these volume abnormalities, particularly if they had experienced an AIDS-defining event. Diffusion tensor imaging, which measures the integrity of white matter fibers, has identified abnormalities of constituents of these fibers in both diseases. Again, people with HIV infection plus alcoholism show the greatest abnormalities, particularly those with a history of an AIDS-defining event. Magnetic resonance spectroscopy, which assesses the levels of brain metabolites and selective neurotransmitters, has revealed different patterns of deficits in biochemical markers of brain integrity in individuals singly affected and a compounding of effects in individuals with both HIV infection and alcoholism. Finally, neuropsychological studies have revealed impairment in selective functions involving working memory, visuospatial abilities, and movement speed that are especially likely to occur in people with comorbid HIV infection and alcoholism. Thus, alcoholism is a major risk factor for development of neuropathology and its functional sequelae in HIV-infected people.

The incidence of alcohol abuse and dependence among individuals infected with human immunodeficiency virus (HIV) is high. Those with concurrent HIV infection and alcohol abuse are at risk of poorer clinical outcome for a variety of reasons that are described in detail elsewhere in this issue of *Alcohol Research & Health*. HIV infection and excessive alcohol use each have specific negative effects on central nervous system (CNS) structure, chemistry, and function, some of which are unique to each disease and some of which are shared. Thus, the brains of people with a history of excessive alcohol consumption are particularly vulnerable to further insult when newly exposed to the HIV virus. Continued excessive drinking can exacerbate the new liability from HIV infection, especially in those who develop an AIDS-defining event, such as severely low numbers of immune cells (i.e., CD4^+^ T-cell count under 200/mm^3^) targeted by HIV infection. The many factors affecting this dynamic interaction of effects of alcohol abuse and HIV infection on the brain are challenging to identify and disentangle but are likely to include the following: general health; cognitive status; age at HIV infection; length and severity of alcohol history *before* HIV infection; quantity, frequency and pattern of alcohol use *after* HIV infection; the lag between the HIV diagnosis and start of antiretroviral treatment; diligence in medication compliance; and whether other drugs of abuse are used.

One method for identifying how the effects of excessive alcohol use and HIV infection interact in the brain is to follow people with both active alcoholism and HIV infection and to compare changes in their cognition and brain status over time with changes in individuals with only one condition and control subjects with neither condition; this is known as a four-group comparison. Given the human condition, such studies (especially longitudinal ones entailing repeated examination of the same individuals over an extended time) are necessarily naturalistic and not ethically or practically amenable to formal experimental control over relevant variables, such as alcohol or illicit drug use, nutrition, medication, or risky behavior. Nonetheless, cross-sectional comparisons (i.e., examination at a single time of many different individuals) between the four groups can provide provisional answers to the question of how and in what way HIV infection exacerbates the damage caused by pre-existing alcoholism or, conversely, how excessive alcohol use exacerbates the damage caused by pre-existing HIV infection. This analysis is best performed with a study sample in which the comorbid group is comparable in severity of their HIV disease to HIV-only participants and in severity of alcohol disease to alcohol-only participants (see Rosenbloom et al. 2007*b*). If alcohol exacerbates the effects of HIV infection, the comorbid group would be predicted to demonstrate greater deficits than either single-diagnosis group, especially for those measures that are affected by both diseases. Such compounded effects could be additive in some cases and synergistic in others.

This brief review summarizes evidence of regional CNS damage from neuroimaging studies using noninvasive magnetic resonance (MR) technologies for in vivo examination of brain structure (conventional magnetic resonance imaging [MRI]), white matter fiber integrity (MR diffusion tensor imaging [DTI]), and brain chemistry (MR spectroscopy [MRS]) in people comorbid for alcoholism and HIV infection compared with single-diagnosis groups and unaffected control subjects. (For more information on these technologies, see the textbox.) Overviews of these methods also are detailed elsewhere (Adalsteinsson et al. 2002; Meyerhoff 2001). Also summarized is evidence from neuropsychological studies examining cognitive and motor functions in the four study groups.

## MRI and Brain Macrostructure

Conventional structural MRI studies collect high-resolution images of the whole brain and permit measurement of the size and shape of brain structures and some aspects of brain tissue integrity.

### Findings From Subjects With Alcoholism

Structural MRI studies of uncomplicated alcoholism typically report increased size of the cerebrospinal fluid-filled spaces in the brain (e.g., ventricular enlargement) as well as gray matter and white matter volume shrinkage (Fein et al. 2002; Gazdzinski et al. 2005; Pfefferbaum et al. 1992, 2001), most prominent in frontal cortical regions (Cardenas et al. 2007; Pfefferbaum et al. 1997) and subcortical and cerebellar structures (Makris et al. 2008; Sullivan and Pfefferbaum 2009). These volume abnormalities may be dose related (Cardenas et al. 2005; Pfefferbaum et al. 1998), at least partially reversed with extended sobriety (Carlen et al. 1978; O’Neill et al. 2001; Pfefferbaum et al. 1995; Rosenbloom et al. 2007*a*), and return with relapse to drinking (O’Neill et al. 2001; Pfefferbaum et al. 1995). Damage to the frontal cortex may contribute to impairment in problem solving and inhibiting undesirable and risky behaviors, whereas damage to the cerebellum likely contributes to the difficulties in balance and gait that frequently are observed in alcoholics.

**Studying the Brain in the Living Body Using Magnetic Resonance Technology**

Several noninvasive technologies can be used to study the structure and function of the living brain. Some of these tools are based on the concept of magnetic resonance (MR)—the observation that positively charged particles (i.e., protons) in certain atoms can be detected by placing the tissues containing these atoms in a magnetic field and recording the results. There are several MR-based technologies available, including MR imaging (MRI), diffusion tensor imaging (DTI), and MR spectroscopy (MRS) (More detailed overviews of these methods have been provided by Adalsteinsson et al. 2002; Bigler 1996; Meyerhoff 2001; Rosenbloom and Pfefferbaum 2008.)

MRI detects protons in hydrogen atoms contained in water and fat, two of the most common components of the body. Protons behave differently whether they are incorporated in water molecules or in fat molecules, and these differences can be visualized as differences in intensity on the MRI. Accordingly, tissues with high water content have a different image intensity compared with tissues with a high fat content. These image intensity differences can be used to determine the size, shape, and tissue composition (i.e., gray matter versus white matter) of the brain and thus allow for visualization of gross (i.e., macrostructural) brain neuroanatomy.

DTI makes use of the fact that the water molecules in the brain always are moving, a process known as diffusion. When water molecules are unconstrained by any barriers (e.g., cell membranes), they can move freely in all directions; this movement is random and called isotropic. In tissue with a regular microstructure, such as brain white matter, however, the movement of the water molecules is constrained and follows the orientation of organized microstructure. This movement is called anisotropic. The degree of anisotropy (i.e., fractional anisotropy) and, conversely, of freedom of movement (i.e., diffusivity) can be measured and visualized to determine white matter microstructure. Thus, high fractional anisotropy and low diffusivity, with certain exceptions, reflect healthy white matter.

MRS is based on similar principles as MRI but measures the levels of different metabolites in body tissues rather than the distribution of water and fat. Thus, MRS provides biochemical rather than structural information about brain tissue. Moreover, the results of an MRS analysis are not presented as an anatomical image of brain tissue but as a spectrum with peaks (i.e., magnetic resonances of selective chemicals) indicating the identity and amount of neurotransmitters and neurometabolites detected. Differences in these peaks measured in patients and controls can indicate abnormalities in the metabolism of the cells studied.

### Findings From Subjects With HIV Infection

Structural MRI studies of HIV infection report little to no brain volume abnormalities in asymptomatic HIV-infected individuals, but there is substantial neuropathology in symptomatic cases (i.e., those with clinically detectable motor, cognitive, or behavioral symptoms) (Aylward et al. 1995; Jernigan et al. 1993). Brain abnormalities include ventricular enlargement, widespread tissue shrinkage, frontal white matter and caudate volume loss (Aylward et al. 1995; Di Sclafani et al. 1997; Stout et al. 1998), and thinning of the cortex (Thompson et al. 2005) and corpus callosum (Pfefferbaum et al. 2006*c*). The extent of tissue volume shrinkage or ventricular expansion increases with advancing clinical stage, as determined using Centers for Disease Control and Prevention criteria (Di Sclafani et al. 1997; Thompson et al. 2006), and is related to presence or worsening of cognitive deficits (Heaton et al. 1995; Heindel et al. 1994; Stout et al. 1998).

### Findings From Subjects With HIV Infection Plus Alcoholism

**Figure 1:**
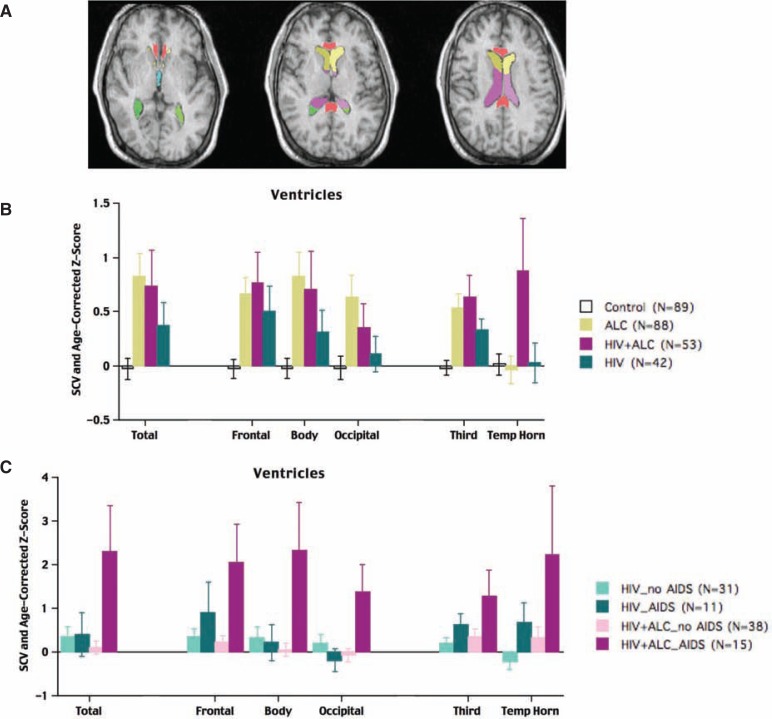
Magnetic resonance imaging (MRI) of the cerebrospinal fluid-filled spaces (i.e., ventricles) in the brain. **A)** Three MRI images representing three horizontal cross-sections of the brain, from lower (left) to higher (right) brain regions. The locations of the various ventricles are indicated by colors and are divided into frontal regions (yellow and mustard); body; or middle regions (magenta) and posterior (i.e., occipital) regions (green); the third ventricle (visible only on the lowest slice) is colored turquoise. The temporal horn, (quantified in **B**) is not visible in these three slices. Also highlighted on this image are the anterior and posterior sections of the corpus callosum (colored in salmon), the white matter structure fully illustrated in the sagittal view shown in Figure 2. **B)** Volumes of the total ventricular system and regions of the ventricular system of control subjects, patients with alcoholism only, patients with human immunodeficiency virus (HIV) and alcoholism, and patients with HIV infection only. Higher scores represent greater abnormality because enlarged ventricular volumes can reflect brain tissue shrinkage. All three patient groups had larger ventricular volumes than the control subjects; however, the ventricular volumes of the two groups that included alcoholic patients showed the greatest enlargement. **C)** Total and regional ventricular volumes for HIV-infected patients with and without acquired immune deficiency syndrome (AIDS) and with and without alcoholism. HIV-infected patients with AIDS and alcoholism had by far the largest ventricular volumes. NOTE: The volumes of the three patient groups were adjusted for variation in head size and age in the control subjects. The bars and error bars signify the means ± standard error for each group for each measure.

**Figure 2:**
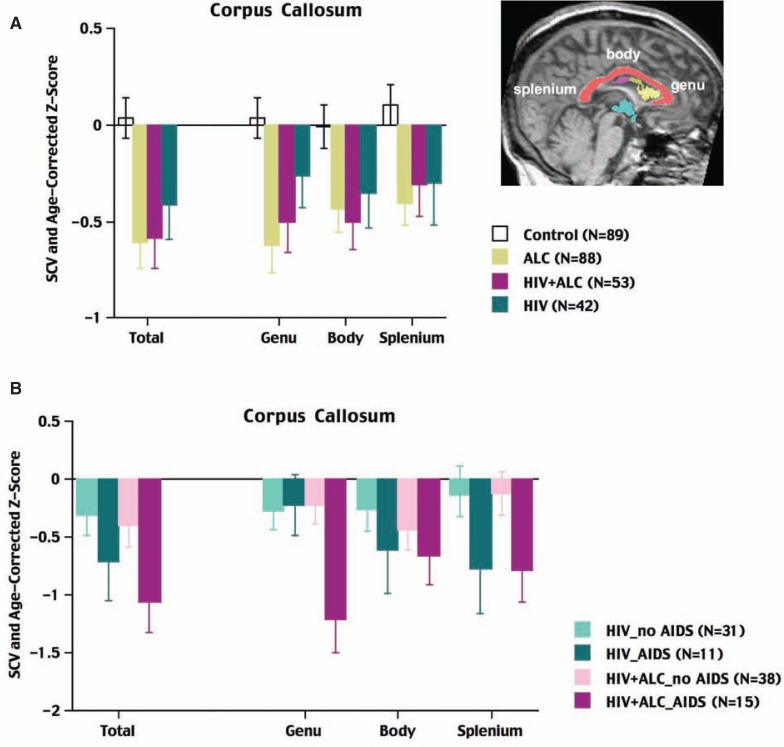
Magnetic resonance imaging (MRI) of the corpus callosum of the brain. MRI represents a sagittal section along the midline of a brain, with the corpus callosum—the bundle of fibers that connects the right and left halves (i.e., hemispheres) of the brain—displayed in salmon color. Also highlighted in this image are views of the frontal (yellow) and middle (magenta) regions of the ventricles as well as the third ventricle (turquoise) shown in Figure 1A. **A)** The volumes of the total and regional divisions of the corpus callosum in control subjects, patients with alcoholism only, patients with human immunodeficiency virus (HIV) and alcoholism, and patients with HIV infection only. All three patient groups had smaller volumes of the corpus callosum than the control group, but the abnormalities of the two groups that included alcoholic patients were especially prominent. **B)** Total and regional volumes of the corpus callosum for HIV-infected patients with and without a history of acquired immune deficiency syndrome (AIDS) and with and without comorbid alcoholism. HIV-infected patients with AIDS and alcoholism showed the greatest callosal abnormalities. NOTE: The volumes of the three patient groups were adjusted for variation in head size and age of the control subjects; the bars and error bars signify the means ± standard error for each group for each measure.

In a structural MRI study using the four-group method, there was a graded pattern of ventricular enlargement. The total ventricular system of the uncomplicated HIV group was larger than that of controls, the HIV group comorbid with alcoholism was yet larger, and the alcoholism group was the largest of the three patient groups. The pattern of callosal thinning showed a similar graded effect. In the HIV group comorbid for alcoholism, larger volumes of the third ventricle correlated with lower CD4^+^ cell counts. When the HIV group was divided according to whether participants had ever met criteria for AIDS (i.e., an AIDS-defining event or low CD4^+^ T-cell counts ≤200/mm^3^), alcohol abuse was shown to have a dramatically compounding effect in those with HIV+AIDS but not in those with HIV infection who had not suffered an AIDS-defining event (figures 1 and 2). The effect was especially notable in the genu of the corpus callosum and the frontal and body regions of the lateral ventricles (Pfefferbaum et al. 2006*c*).

## DTI and Brain White Matter Microstructure

DTI yields measures of the microstructural integrity of white matter (Le Bihan 2003) and provides a method to ascertain how various pathologies affect the microstructure of the brain. Studies typically examine DTI metrics in a defined region of white matter, such as the corpus callosum or centrum semiovale, or can examine tissue integrity along a statistically extracted fiber bundle, a procedure known as quantitative fiber tracking (Gerig et al. 2005). Although fiber tracking does not actually identify anatomically specific fibers or fiber bundles, tractography can provide a statistical and graphical representation along the length of fiber bundles (Mori and van Zijl 2002; Rosenbloom and Pfefferbaum 2008). DTI’s sensitivity to detection of disruption of microstructural integrity of white matter may provide an early indication of the potential for HIV-associated dementia (Berger and Avison 2001) and signs of insult from co-existing alcoholism (Pfefferbaum et al. 2007*a*).

### Findings From Subjects With Alcoholism

Among alcoholics, DTI has revealed evidence for disruption of microstructural constituents of white matter even in regions appearing normal on conventional structural imaging (Pfefferbaum et al. 2006*a*; Pfefferbaum and Sullivan 2002). Chronic alcohol abuse also disrupts microstructure in localized regions of white matter, such as the corpus callosum, and widely distributed regions throughout the centrum semiovale (Pfefferbaum et al. 2006*a*, *b*) or in right hemisphere white matter tracts linking prefrontal and limbic systems (Harris et al. 2008). A fiber tracking study examining the integrity of a broad range of white matter tracts across the brain found that frontal and superior sites (frontal forceps, internal and external capsules, fornix, and superior cingulate bundle and longitudinal fasciculi) showed greatest abnormalities in alcoholics relative to controls, while more posterior and inferior bundles were relatively spared (Pfefferbaum et al. 2009*b*). Poor performance on tests of working memory, visuospatial ability, and gait and balance have been linked to compromised white matter microstructure and amount of alcohol drunk in a lifetime in alcoholics (Pfefferbaum et al. 2006*a*). Disruption of the myelin sheaths may contribute to slowed motor performance by alcoholics (Pfefferbaum et al. 2009*b*).

### Findings From Subjects With HIV-Infection

Examination of HIV-infected individuals with DTI has revealed microstructural abnormalities of the genu and splenium of the corpus callosum and frontal and parietal subcortical white matter related to disease severity (Filippi et al. 2001). Signs of compromised integrity also can be found in regions of white matter appearing normal on conventional MRI (Stebbins et al. 2007). DTI studies have found signs of compromised white matter integrity in normal-appearing periventricular white matter and the corpus callosum that correlated with low CD4^+^ counts and high viral loads in HIV-infected subjects (Filippi et al. 2001).

Fiber tracking analysis revealed that posterior but not anterior corpus callosum fibers are affected in nondemented HIV-infected patients (Pfefferbaum et al. 2009*a*). Altered DTI metrics, primarily reflecting axonal compromise, have been found in the following fibers bundles:

- The internal and external capsules, which are fiber bundles that course through the striatum and connect corticospinal regions and the cerebral cortex;
- The superior and inferior cingulate bundles that link frontal and subcortical structures; and
- The posterior sectors of the corpus callosum, which links posterior parietal and temporal regions of the left and right hemispheres.

By contrast, pontocerebellar projection fibers were particularly resistant to HIV effects, as were commissural fibers coursing through premotor and sensorimotor callosal sectors (Pfefferbaum et al. 2009*a*). Cognitive impairment has been related to white matter degradation and is more severe in patients whose HIV infection has progressed to AIDS (Gongvatana et al. 2009). Similarly, slower speed on tests of finger movement and eye-hand coordination is related to degradation of posterior callosal fibers (Pfefferbaum et al. 2007*b*).

### Findings From Subjects With HIV-Infection Plus Alcoholism

**Figure 3:**
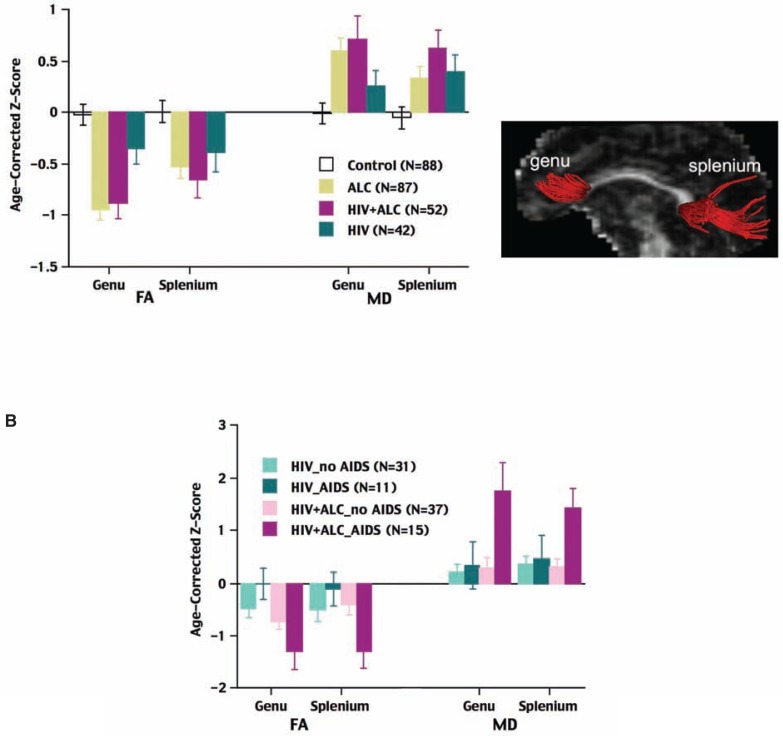
Example of diffusion tensor imaging (DTI) fiber tracking of the frontal (i.e., genu) and posterior (i.e., splenium) regions of the corpus callosum displayed in red. **A)** Representation of the differences in two DTI measures in patients with alcoholism alone, human immunodeficiency virus (HIV) infection alone, HIV and alcoholism, and control subjects with neither condition. Fractional anisotropy (FA)^*^ and mean diffusivity (MD) are measures reflecting the integrity of a white matter fiber; lower scores for FA and higher scores for MD indicate fiber integrity compromise. All three patient groups had lower callosal FA and higher MD than the control subjects, but the genu abnormalities of the two alcohol groups were statistically significant. **B)** FA and MD values in the HIV-infected patient groups divided according to their history of acquired immune deficiency syndrome (AIDS). HIV-infected patients with AIDS and alcoholism showed the greatest callosal abnormalities.

A DTI fiber tracking study examined the effects of HIV-alcoholism comorbidity in the four-group model (Pfefferbaum et al. 2007*a*). The microstructural integrity of fiber bundles coursing through the genu and splenium of the corpus callosum showed evidence of severe compromise in those with alcoholism whose HIV infection history included an AIDS-defining event (figure 3). Measures of DTI provided indirect support for greater disruption of the myelin sheath covering the axon (i.e., fatty cell body that insulates the axon and promotes speed of communication between cells) in alcoholism (Pfefferbaum et al. 2009*b*) than of the axon itself as observed in HIV infection (Pfefferbaum et al. 2009*a*). The functional significance of these callosal fiber tracking measures in the AIDS+alcoholism group was demonstrated by analyses showing that greater degradation of fiber integrity was associated with slower and less dexterous manual motor performance and greater postural instability while standing (Pfefferbaum et al. 2009*b*).

## MRS and Brain Chemistry

**Figure 4:**
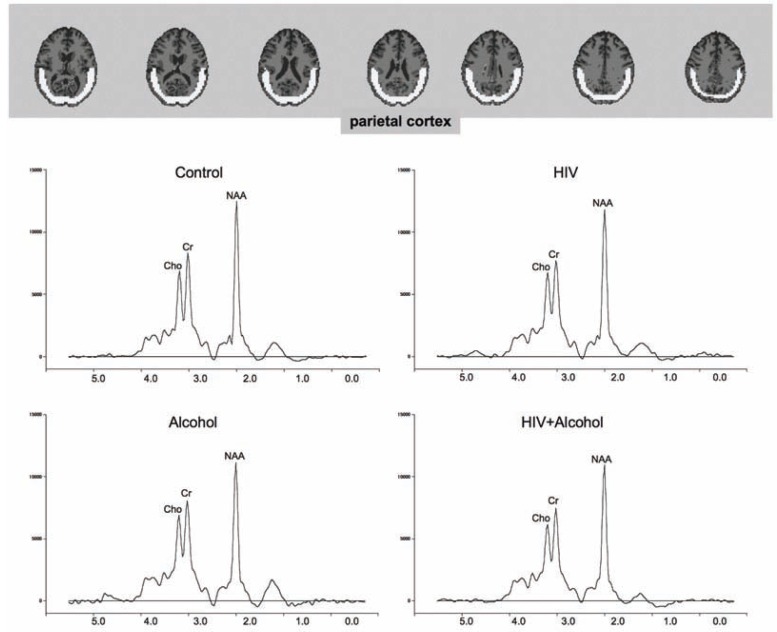
Magnetic resonance spectroscopy (MRS) in a parieto-occipital cortical sample of the brain. Images show a representative example (34-year-old man with human immunodeficiency virus [HIV] infection and alcohol dependence) of the parietal-occipital cortical region of interest (white) used in MRS imaging metabolite quantification. MRS spectra represent analyses of various brain metabolites in people with HIV infection alone, alcoholism alone, comorbid HIV infection and alcoholism, and control subjects with neither condition. The peak representing the metabolite *N*-acetyl-aspartate (NAA) shows a significant deficit in the HIV plus alcoholism group compared with the other groups.

MRI and DTI are imaging modalities based primarily on the detection of water protons, their immediate environment, and their mobility. Other constituents, including selective metabolites and neurotransmitters, also are present on the proton spectrum but in smaller quantities than water, yet visible using other MR technologies, such as MR spectroscopy (MRS) and MR spectroscopic imaging (MRSI) (Adalsteinsson et al. 2002; Meyerhoff 2001). A substantial body of research using proton MRS and MRSI (also known as chemical shift imaging [CSI]) has revealed declines in N-acetyl aspartate (NAA), a marker of living neurons, in short-term abstinent alcoholics, with change toward normalization of NAA in longer-term abstinent alcoholics (Durazzo et al. 2006). Use of proton MRS in HIV infection has provided evidence for metabolite abnormalities notable in the basal ganglia, even early in the course of HIV infection (Chang et al. 2008). A proton MRSI study of combined disease effects on the parietal-occipital cortex indicated that only the comorbid (HIV+alcoholism) group was affected, exhibiting a significant deficit in NAA (figure 4). Although neither HIV infection nor alcoholism alone resulted in such a deficit, each disease carried a liability that put dually affected individuals at a heightened risk of neuronal compromise (Pfefferbaum et al. 2005).

In addition to proton (^1^H) spectroscopy, other resonances (e.g., carbon [^13^C] and phosphorous [^31^P]) that provide unique brain metabolic information can be studied, albeit with difficulty because of their low spectral signal (i.e., low signal-to-noise ratio). A ^31^P MRS study (see Meyerhoff 2001) noted a cumulative but not interactive or synergistic effect of HIV infection and alcoholism. The symptomatic HIV group and the alcoholic group both had low concentrations of the brain energy metabolites associated with cell membrane breakdown (phosphodiester) and building (phosphocreatine) in superior white matter, and individuals with both conditions had augmented metabolite deficits (Meyerhoff et al. 1995).

## Neuropsychological Functioning in HIV–Alcoholism Comorbidity

An extensive literature is available that describes patterns of neuropsychological impairment associated with alcoholism (for reviews, see Oscar-Berman and Marinkovic 2007; Sullivan 2000) and HIV infection (see Special Section, “HIV/NeuroAIDS” in *Neuropsychology Review*, June 2009). Significantly fewer studies have considered the effects of HIV+alcoholism comorbidity on cognitive and motor functioning, but an emerging literature indicates that such comorbidity exerts selective rather than simply global adverse effects on component processes of functions. To date, functions examined and found to be disproportionately affected by the combined diseases include speeded performance involving finger movements and eye-hand coordination (i.e., visuomotor speed) (Fama et al. 2007; Rothlind et al. 2005), sustained attention (Sassoon et al. 2007), conflict resolution and attentional allocation (Schulte et al. 2008), associative learning (Sassoon et al. 2007), immediate episodic memory (Fama et al. 2009), and speeded finger movements (Fama et al. 2007).

Brain regions likely contributing to performance on these tasks and known to be disrupted by alcoholism or HIV infection independently or by their combination include the following:

- The frontostriatal system, which is required for working memory, psychomotor speeded activities, and conflict resolution;
- The frontoparietal system, which is the basis of selective attentional and visuospatial networks; and
- The frontocerebellar system, which contributes to verbal and spatial working memory, smooth and speeded motor function, and postural stability.

**Figure 5:**
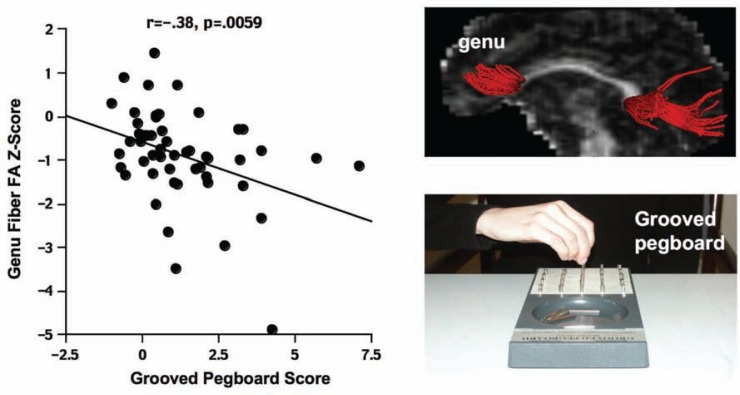
Low fractional anisotropy (FA) measured with diffusion tensor imaging (DTI) typically indicates compromised fiber integrity. The grooved pegboard test (bottom right image) requires participants to insert keyed pegs into keyed holes as quickly as possible. The data scatter plot (left panel) displays a correlation indicating that lower FA in the frontal region (i.e., genu) of the corpus callosum (top right image)—was predictive of greater length of time taken to complete the pegboard test.

A useful neuropsychological model to explain some of the results is based on the observation that alcoholism has a frontocerebellar substrate of dysfunction, whereas HIV infection has a frontostriatal substrate of dysfunction. Tasks invoking both of these circuits and the tracts linking these regions (figure 5) may be at particular at risk of disruption from both conditions (Pfefferbaum et al. 2002).

## Conclusion

The combined effects of alcoholism and HIV infection on brain structure, chemistry, and function are most devastating in individuals who have experienced an AIDS-defining event. This negative synergism suggests that alcohol is particularly toxic to the HIV-infected brain after a certain stage of the disease; similarly, the alcoholic brain is particularly susceptible to the effects of AIDS. These effects place dually affected individuals at compounded risk for brain functional and structural insult even prior to developing clinically defined dementia, thereby highlighting the relevance of examining groups affected by both conditions (see Fein et al. 1995; Meyerhoff 2001; Pfefferbaum et al. 2007*a*; Pfefferbaum et al. 2002; Rothlind et al. 2005). The substantial effect of the alcoholism–AIDS interaction on ventricular volumes and callosal size and microstructure, in the context of the modest changes observed in non-AIDS, non-alcohol–abusing HIV-infected individuals, highlights the need to consider alcohol use disorders as a major risk factor for developing neuropathology among HIV-infected persons (Pfefferbaum et al. 2007*a*). Further, the high prevalence of alcoholism in HIV-infected individuals and the interfering effect of alcohol abuse on HIV pharmacological response and therapy compliance underscore the need to recognize the independent and synergistic contributions of each condition to brain structure and function (Pfefferbaum et al. 2006*c*). The extent to which the deficits in patients with comorbid HIV infection and alcoholism are reversible with abstinence from alcohol and careful adherence to antiretroviral treatments requires investigation using longitudinal study.
